# Enhancing NIR Shielding Properties of Au/CsWO_3_ Composite via Physical Mixing and Solvothermal Processes

**DOI:** 10.3390/ma17112746

**Published:** 2024-06-05

**Authors:** Chanakarn Piwnuan, Chivarat Muangphat, Jatuphorn Wootthikanokkhan

**Affiliations:** Materials Technology Program, School of Energy, Environment and Materials, King Mongkut’s University of Technology Thonburi, 126 Pracha Uthit Rd., Bangmod, Thung Khru, Bangkok 10140, Thailand; chanakarn.piwn@kmutt.ac.th (C.P.); chivarat.mua@kmutt.ac.th (C.M.)

**Keywords:** gold/CsWO_3_ nanorods, composites, NIR shielding, plasmonic coupling, localized plasmon resonance

## Abstract

This research aims to enhance the near-infrared (NIR) shielding ability of cesium tungsten bronze (CsWO_3_) by increasing the spectral absorption in this region through the incorporation of gold nanorods (Au_NR_). Two approaches were used to prepare the composite materials: physical mixing and solvothermal process. The effects of gold nanorods content on the crystalline size, particle size, shape, and optical properties of the composite were investigated systematically using DLS, TEM, XRD, and UV–Vis spectroscopy techniques, respectively. The physical mixing process synergizes Au_NR_ and CsWO_3_ into a composite which has better NIR absorption than that of neat Au_NR_ and CsWO_3_ nanorods. A composite with 10 mol% of Au_NR_ shows the highest NIR absorption ability due to the surface plasmon resonance and energy coupling between Au and CsWO_3_. With the solvothermal process, the CsWO_3_ nanorods grow up to 4–7 microns when the Au_NR_ content increases to 0.8 mol% due to the incorporation of the Au atoms. The microsized CsWO_3_ rods have superior NIR shielding property compared to other conditions, including the Au_NR_+CsWO_3_ nanocomposite with 10 mol% of Au_NR_ from the physical mixing process.

## 1. Introduction

The 2022 United Nations Environment Program Report stated that energy consumed in buildings accounted for around 34% of global energy consumption in 2021 [1], marking a 4% increase from 2020. The major energy-consuming activities in buildings include heating, cooling, ventilation, lighting, and cooking. Energy consumption for cooling has increased due to the increase in global temperature [2]. During the day, indoor temperatures are elevated due to near-infrared light, which constitutes about half of solar energy. NIR light generates heat rays that can penetrate buildings, leading to increased energy consumption for air conditioning and subsequent rise in electricity demand.

In order to improve energy efficiency in buildings without losing human thermal comfort, the transfer of heat through the building envelope should be decreased. In this regard, NIR shielding materials have attracted more attention in recent years [3]. In recent years, transparent conductive oxides such as indium tin oxide (ITO) [4,5], aluminum zinc oxide (AZO) [6], and antimony tin oxide (ATO) [7,8,9] have been developed for use as NIR shielding materials, owing to their high absorbance and/or reflectance in the NIR region. However, ITO and AZO only effectively shield the NIR radiation over the wavelength above 1500 nm [5,6], whereas ATO shows poor NIR shielding performance in the wavelength ranging between 700 and 1300 nm [3].

Cesium tungsten bronze (Cs_x_WO_3_) with a hexagonal structure is a promising material for its interesting properties, including optical, heat-shielding, and electrical properties [10,11,12,13]. It is particularly promising when compared to other types of tungsten bronze such as Na_x_WO_3_ [14], K_x_WO_3_ [15], and Rb_x_WO_3_ [16], especially due to its ability to suppress spectral transmittance in the NIR region [17].

To enhance the optical properties of Cs_x_WO_3_, several preparation methods have been studied to control the size and morphology of the material. For example, Cs_x_WO_3_ with irregular particles in the range of 0.1–5.0 μm have been synthesized by solid state reaction and the hydrothermal method [14,18,19]. These methods demonstrated poor control over the size of Cs_x_WO_3_ nanoparticles, leading to low performance in terms of NIR shielding ability and visible light transmittance. Recently, Guo et al. [20] synthesized Cs_x_WO_3_ nanorods with controlled dimensions (approximately 15 nm width and 50 nm length) via solvothermal process. They demonstrated that NIR shielding ability and visible light transparency of the nanorod were superior to those of irregular Cs_x_WO_3_ particles. However, the NIR shielding ability of Cs_x_WO_3_ over the wavelength ranging from 700 nm to 1100 nm has yet to be improved [20].

Several studies have paid attention to improve the NIR shielding ability of CsWO_3_ by mixing or doping it with other materials. Xu et al. [21] synthesized CsWO_3_ by hydrothermal process and mixed it with ATO nanoparticles via a ball milling process. The CsWO_3_/ATO composite with a weight ratio of 1/1 exhibited an increase in NIR absorption percentage of up to 90%, compared with neat CsWO_3_ particles. In addition, Wu et al. [22] proposed CsWO_3_/ZnO composites to be used as a smart coating. The CsWO_3_ was prepared by a solvothermal process, and then the ZnO was introduced via a mild chemical process. When the mass ratio of CsWO_3_/ZnO was 1/1, the composite coating possessed a high visible light transmittance of over 80% and great NIR shielding ability. Alternatively, the fluorine-doped CsWO_3_ was synthesized by adding hydrofluoric acid into the mixture of CsWO_3_ solution, prior to carrying out the hydrothermal process [23]. As a result, the Cs_0.33_WO_3-x_F_x_ film with an F/W molar ratio of 0.45/1 exhibited a higher NIR shielding ability of 90% compared with the undoped Cs_0.33_WO_3_ film.

In this study, attempts were made to enhance the NIR shielding ability of CsWO_3_ by introducing gold nanorods (Au_NR_) into the system. The absorbance spectrum of Au can be tuned by controlling the size, shape, and structure of materials [24]. The absorption peak of gold nanoparticles can be shifted from visible region to NIR region (>780 nm) by increasing the aspect ratio of Au_NR_ through localized surface plasmon resonance [25]. This statement was confirmed by several studies, including the study by Makhsin et al., which demonstrated that the absorption peak of Au_NR_ was shifted toward the long wavelength over 700 nm when the aspect ratio of Au_NR_ was larger than 3.5 [26].

**Figure 1:**
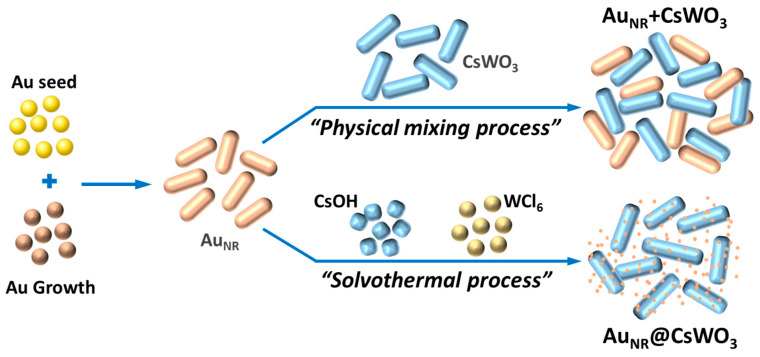
Schematic of preparation of Au_NR/_CsWO_3_ composites via two different approaches.

Despite the above progress and to the best of our best knowledge, the optical properties of a composite system containing a combination of Au_NR_ with CsWO_3_ have not been studied and reported in the open literature. This work aimed to investigate the effects of the Au_NR_ content on the morphology, crystal structure, and optical structures of the resulting composite materials. Two different approaches were used to prepare the composites: a physical mixing process and an in situ mixing of Au_NR_ with CsWO_3_ via a solvothermal process, as illustrated in Figure 1. It was found that, by properly controlling the amount of Au_NR_, the composite materials prepared via the latter approach could exhibit enhanced optical properties.

## 2. Materials and Methods

### 2.1. Materials

Tungsten hexachloride (WCl_6_), cesium hydroxide monohydrate (CsOH·H_2_O), gold (III) chloride trihydrate (HAuCl_4_·3H_2_O), sodium borohydride (NaBH_4_), and cetyltrimethylammonium bromide (CTAB) were purchased from Sigma-Aldrich Pty Ltd. (St. Louis, MO, USA). Silver nitrate (AgNO_3_), ethanol, acetic acid, and hydrochloric acid were purchased from Merck Co. Ltd. (Burlington, MA, USA). Ascorbic acid was acquired from Tokyo Chemical Industry Co., Ltd. (Tokyo, Japan). All these chemicals were of analytical grade and used as purchased without further purification. Deionized water was required in all experiments.

### 2.2. Synthesis of CsWO_3_ Nanorods

The rod-like CsWO_3_ nanoparticles were synthesized via a water-controlled release solvothermal process [27]. In a typical experiment, 1.0 mmol of WCl_6_ powder and 0.5 mmol of CsOH·H_2_O powder were dissolved in 40 mL of absolute ethanol with constant stirring. Then, 10 mL of acetic acid was added and mixed into the prepared solution. Next, the solution was transferred into a Teflon-lined autoclave of 100 mL internal volume, followed by solvothermal treatment at 240 °C for 20 h. After that, dark blue powders were collected using centrifugation and washed with deionized water and ethanol three times, then dried at 60 °C for 12 h.

### 2.3. Synthesis of Gold Nanorods (Au_NR_)

Gold nanorods were synthesized via the seed-mediated growth method [28]. First, the seed solution was prepared by dissolving 0.01 M of HAuCl_4_ into 0.1 M of CTAB solution by gently stirring for 5 min. After that, 0.01 M of ice-cold NaBH_4_ solution was added to the solution by vigorously stirring for 2 min. A color change in the solution, from yellow to brownish yellow, should be noticed. This CTAB-stabilized seed solution was kept at room temperature for 2 h.

The growth solution was prepared by mixing 0.1 M of CTAB with 0.006 M of AgNO_3_. Then, 0.01 M of HAuCl_4_ and 1.0 M of hydrochloric acid in aqueous solution were, respectively, added to the growth solution under gentle stirring. A total of 0.1 M of ascorbic acid in aqueous solution was added at once, and the growth solution became colorless. The CTAB-stabilized seed solution (10 µL) was added to the growth solution by gently mixing for 10 s. Then, the solution was kept at room temperature for 18 h. After 18 h, Au_NR_ were centrifuged and washed with deionized water to remove some excess CTAB. The product was resuspended in 5 mL of deionized water and stored at room temperature before use.

### 2.4. Preparation of Au_NR_+CsWO_3_ Composite Materials by Physical Mixing Method

CsWO_3_ powder was added into an aqueous solution of Au_NR_ in a beaker under constant stirring at room temperature for an hour. The molar ratios between Au_NR_ and CsWO_3_ varied from 0.8 to 20 mol%.

### 2.5. Preparation of Au_NR_@CsWO_3_ Composite Materials by Solvothermal Method

Typically, 0.5 mmol of WCl_6_ powder and 0.25 mmol of CsOH·H_2_O powder were dissolved in 20 mL absolute ethanol with constant stirring. Then, 5 mL of acetic acid was added to this solution, followed by adding 3 mL of a solution of Au_NR_ (0.3, 0.6, 1.2, and 2.4 *w*/*v*% in ethanol) under stirring. These values are equivalent to 0.4, 0.8, 1.5, and 3.0 mol% Au_NR_. The solution was transferred into a Teflon-lined autoclave of 100 mL internal volume, followed by solvothermal treatment at 240 °C for 20 h. Next, the product was centrifuged and washed with deionized water and ethanol, respectively, several times. Finally, the product was dried at 60 °C for 12 h.

### 2.6. Characterizations

The X-ray diffraction (XRD) patterns of the CsWO_3_, Au_NR_, and the Au_NR_/CsWO_3_ nanocomposites were recorded by using a Bruker D8 Advance X-ray diffractometer (Bruker, Billerica, MA, USA), using Cu Kα radiation (λ = 1.5406 Å) and 2θ in the range of 20°–90°. The morphological structure of the materials was analyzed by transmission electron microscopy (TEM, Thermo scientific Talos F200X, Waltham, MA, USA) with an EDS attachment. TEM specimens were prepared by placing a drop of aqueous particle solution on a carbon-coated copper grid and then evaporating the solution at room temperature. The particle size distribution of the nanocomposites was measured by dynamic light scattering technique (DLS), using a Zetasizer instrument (Nano-ZS90, Malvern Instruments Ltd., Malvern, UK). The DLS samples were diluted with DI water and then sonicated to suspend the particles before testing. The average hydrodynamic diameter was evaluated by taking an average of 11 runs, with each run having a duration of 10 s. The optical properties of the samples were measured by a UV–Vis spectrophotometer (Thermo Scientific, Genesys 10S). The nanocomposites were suspended in DI water before testing. The absorbance and transmittance measurement of the nanocomposites was investigated over the wavelength ranged between 300 and 1100 nm at room temperature.

## 3. Results

### 3.1. Au_NR_+CsWO_3_ Nanocomposites by Physical Mixing Method

**Figure 2:**
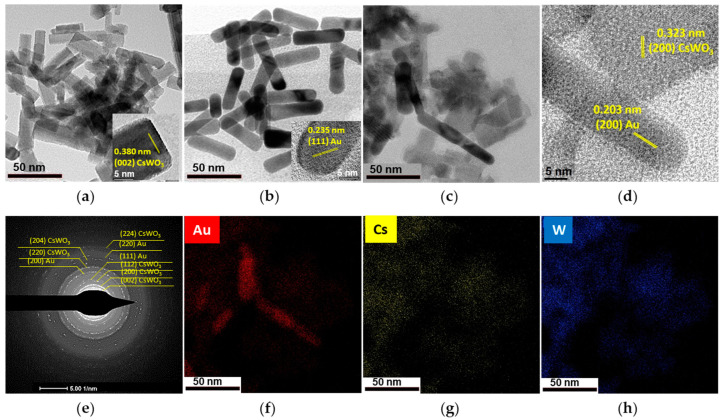
TEM images of (**a**) CsWO_3_ (inset: fringe pattern); (**b**) Au_NR_ (inset: fringe pattern); (**c**) Au_NR_+CsWO_3_ nanocomposites synthesized by physical mixing method with 3.0 mol% Au_NR_; (**d**) fringe pattern of Au_NR_+CsWO_3_ nanocomposites; (**e**) SAED pattern of Au_NR_+CsWO_3_ nanocomposites; and (**f**–**h**) elemental mapping analysis of 3.0 mol% Au_NR_.

The precursors, CsWO_3_ and Au nanorods, were separately synthesized by solvothermal and seed-mediated growth processes, respectively, under the aforementioned conditions. The TEM image in Figure 2a shows the sharp-ended CsWO_3_ nanorod with 13.76 nm width and 48.71 nm length (aspect ratio 3.54), while Figure 2b shows the round-ended Au_NR_ with 15.49 nm width and 52.82 nm length (aspect ratio 3.41).

After combining the precursors by a physical mixing process, the Au_NR_ content of 0.8 and 1.5 mol% was not observed by TEM due to the low Au_NR_ content. However, the appearance of a ring pattern of nanocomposites, corresponding to the Au nanostructure, was detected on the SAED pattern (Figure 2e). When the content of Au_NR_ increased to 3.0 mol%, the size and shape of the round-ended Au_NR_ and sharp-ended CsWO_3_ nanorods were retained, as verified by TEM and elemental mapping analysis in Figure 2c and Figure 2f–h, respectively.

**Figure 3:**
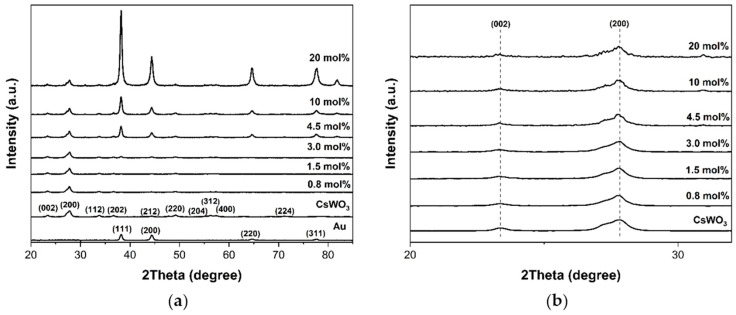
(**a**) XRD patterns of Au_NR_, CsWO_3_, and Au_NR_+CsWO_3_ nanocomposites synthesized by physical mixing method with different Au_NR_ content and (**b**) magnified XRD patterns in the region of 2θ from 20° to 32°.

The crystal structure of the Au_NR_+CsWO_3_ nanocomposite was also examined by XRD (Figure 3). The diffraction peaks at 23.6°, 27.7°, 34.0°, 36.7°, 44.4°, 49.0°, 55.8°, 56.3°, and 57.6° were observed. These correspond to the (002), (200), (112), (202), (212), (220), (204), (312), and (400) planes of the hexagonal structure of cesium tungsten bronze (Cs_0.32_WO_3_), according to the standard ICDD No. 01-086-2578 [29], in both CsWO_3_ and Au_NR_+CsWO_3_ nanocomposites.

Moreover, a diffraction peak was observed at around 38.2°, corresponding to the (111) plane of face-centered cubic (fcc) crystalline lattice of Au, as indicated in JCPDS No. 04-0784. Furthermore, the intensity of Au peaks increased by increasing the content of Au_NR_. Apart from these, no new phase or impurity was observed from the XRD patterns of these nanocomposites. It was also noted that the increase in Au content did not alter the position of peaks at (002) and (200) of Cs_0.32_WO_3_ (Figure 3b).

**Figure 4:**
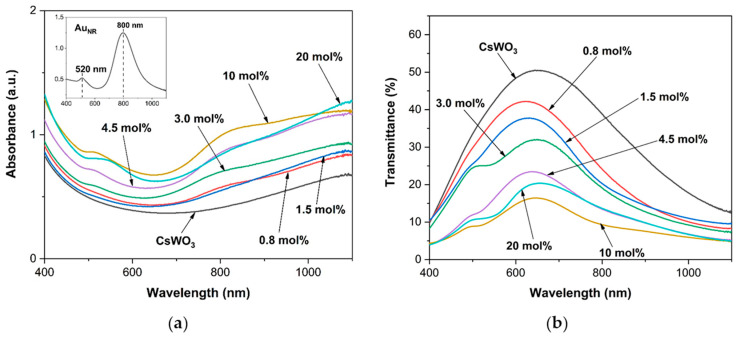
(**a**) Absorbance and (**b**) transmittance spectra of CsWO_3_ nanorods and Au_NR_+CsWO_3_ nanocomposites synthesized by physical mixing method with different Au_NR_ content.

The top-left graph in Figure 4a shows the absorbance spectrum of Au_NR_, which exhibited two resonance modes, a transversal surface plasmon resonance (TSPR) and a longitudinal surface plasmon resonance (LSPR), which in turn determine the optical properties of this rod-shaped nanostructure. The TSPR and LSPR modes correspond to the electron oscillation associated with the short and long axes of the nanorod, respectively. The absorption spectrum of Au_NR_ is related to the aspect ratio and shape, as shown in the TEM images (Figure 2b). The weak absorbance peaks corresponding to the TSPR bands are around 520 nm, while the strong LSPR peaks are around 800 nm, according to the horizontal dimension of Au_NR_.

Figure 4 shows the absorption and transmission spectra of CsWO_3_ nanorods and Au_NR_+CsWO_3_ nanocomposites synthesized by the physical mixing method. The CsWO_3_ nanorods exhibited high transmittance in the visible range (400–780 nm) and low transmittance in the NIR range (780–1100 nm), providing effective infrared shielding. The Au_NR_+CsWO_3_ nanocomposites prepared by physical mixing method demonstrate improved NIR absorbance over CsWO_3_ nanorods. The NIR absorbance increases as the Au_NR_ content increases. Consequently, the visible transmittance of nanocomposites significantly decreases with the higher Au_NR_ content. A 10 mol% Au_NR_ content exhibited the lowest visible light transmittance while offering the highest NIR shielding properties.

**Figure 5:**
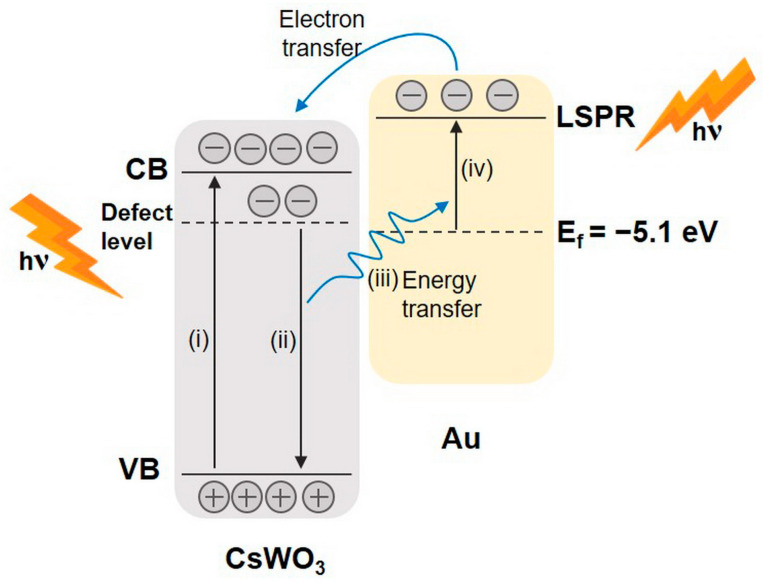
Schematic illustration of the energy coupling and plasmonic hot electron transfer in Au_NR_+CsWO_3_ nanocomposites.

The Au_NR_+CsWO_3_ nanocomposite exhibited unique absorbance spectra, which differs from the optical properties of neat Au_NR_ and CsWO_3_ nanorods. This was ascribed to surface plasmon resonance and energy coupling between Au and CsWO_3_ (Figure 5), which enhanced the NIR absorption by the nanocomposite. Similar behavior has been reported for Au/ZnO and Au/WO_3_ systems [30,31,32,33,34], which demonstrated the mechanism of a sequential energy transfer from ZnO or WO_3_ to Au and electron transfer from Au to ZnO or WO_3_.

As explained in Figure 5, when light is absorbed by electrons on the surface of CsWO_3_, these electrons are excited to the conduction band, while some electrons are trapped at the defect level, which is called small polaron. This contributes to the NIR absorption at 780–1100 nm [35]. Subsequently, the electrons recombine with the holes in the valence band of CsWO_3_, resulting in energy emission. When CsWO_3_ is in contact with Au_NR_, the electrons on the surface of Au_NR_ are elevated to a higher energy state, generating surface plasmon resonance (SPR) by absorbing light. Consequently, the hot energetic Au electrons are transferred to the conduction band of the adjacent CsWO_3_. This additional influx of electrons increases the charge carrier concentration in the conduction band and band structure, leading to an enhanced NIR absorption capability of the nanocomposite.

Moreover, with a higher Au_NR_ content, more hot electrons from Au could be transferred into the conduction band of the adjacent CsWO_3_, resulting in an enhanced NIR absorption ability of the nanocomposite. This synergetic effect between CsWO_3_ and Au_NR_ led to the enhancement in NIR absorption of the physically mixed composite.

### 3.2. Au_NR_@CsWO_3_ Nanocomposites Prepared by the Solvothermal Method

**Figure 6:**
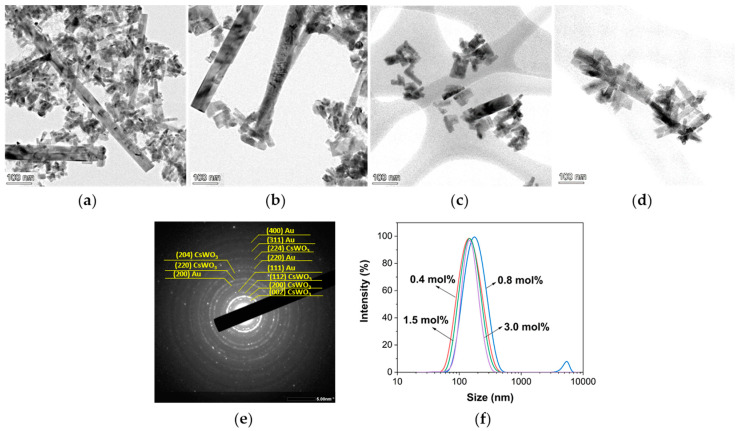
TEM images of Au_NR_@CsWO_3_ nanocomposites with Au_NR_ content of (**a**) 0.4 mol%; (**b**) 0.8 mol%; (**c**) 1.5 mol%; (**d**) 3.0 mol%; (**e**) SAED pattern of Au_NR_@CsWO_3_ nanocomposites; and (**f**) size distribution curve of Au_NR_@CsWO_3_ nanocomposites with different Au_NR_ content.

Figure 6 shows TEM images of the Au_NR_@CsWO_3_ nanocomposite with various Au_NR_ contents. At the lowest Au_NR_ content (0.4 mol%), the sample contains a mixture of rod-shaped CsWO_3_ with two different longitudinal sizes (Figure 6a). Measurement using ImageJ software (version 1.53v) revealed that the majority of the Au_NR_@CsWO_3_ nanocomposites were around 12–15 nm in width and 50–100 nm in length, while a smaller number were approximately 40–80 nm in width and 0.5–1.2 µm in length.

When the Au_NR_ content increased to 0.8 mol%, the size of rod-shaped CsWO_3_ increased in both width and length. Most of the CsWO_3_ nanorods were approximately 16–60 nm in width and 70–600 nm in length, while around 5% were exceptionally long, measuring roughly 60–100 nm in width and 4–7 µm in length. This observation aligned with the DLS results shown in Figure 6f. For Au_NR_ contents of 1.5 and 3.0 mol%, the CsWO_3_ nanorods were approximately 60–400 nm in length, and the longer micron-sized nanorods were no longer observed (Figure 6c,d). This suggests that the increase in the Au_NR_ content led to a shift in the size distribution of the CsWO_3_ nanorods, favoring shorter lengths.

Although the Au_NR_ could not be directly observed in the nanocomposites through TEM imaging, SAED pattern revealed the presence of a ring pattern corresponding to the Au nanostructure (Figure 6e). 

**Figure 7:**
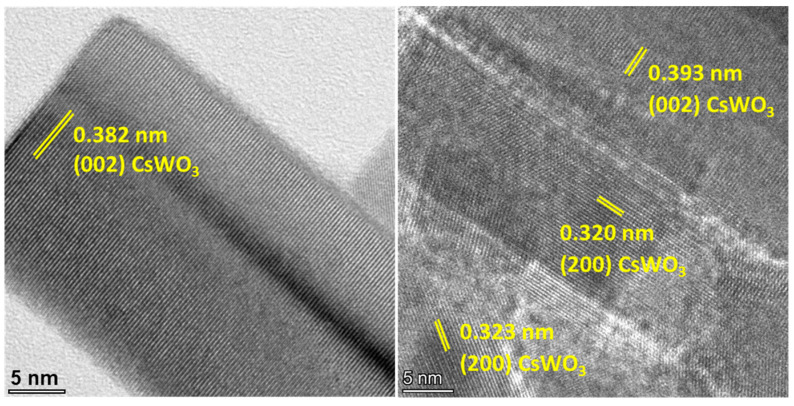
HRTEM images of Au_NR_@CsWO_3_ nanocomposites with 0.8 mol% Au_NR_.

The high-resolution TEM image shows that the lattice fringe spacing of the (002) crystal plane is present at the end of the CsWO_3_ nanorods, while the (200) plane is oriented perpendicular to the nanorod axis. This suggests that there are two possible growth orientations for the nanorods: along the [002] direction and the [200] direction, as depicted in Figure 7. This result is consistent with previous work by Guo et al. [18], which proposed that the (002) plane parallel to the nanorod is the primary growth direction for CsWO_3_ nanorods.

**Figure 8:**
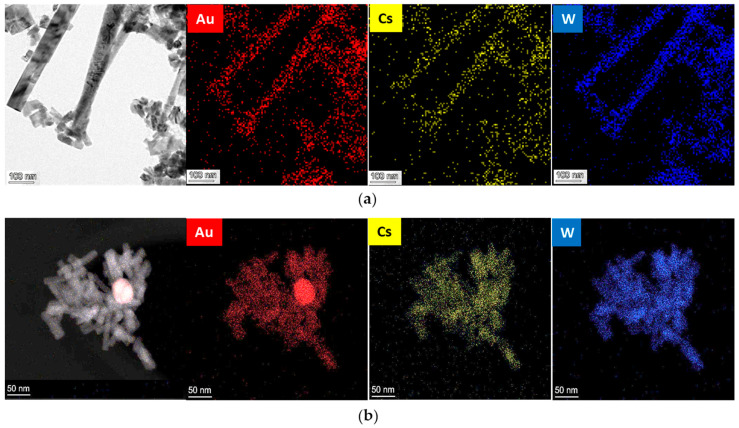
Elemental mapping analysis of (**a**) 0.8 mol% Au_NR_ at 8000x magnification and (**b**) 3.0 mol% Au_NR_ at 250 kx magnification.

Although the Au_NR_ content of 0.8 mol% was not directly observable using TEM, the SAED analysis revealed a ring pattern corresponding to the face-centered cubic (FCC) structure of Au. Elemental mapping further indicated that the Au phase was homogeneously distributed within the CsWO_3_ nanorods (Figure 8a). This suggests that the high temperature (>250 °C) in the solvothermal process may have caused morphological changes to the original Au_NR_, leading to decomposition into atoms and dispersion in the solution, as reported by Petrova et al. [36], where some of these Au atoms may further attach to growing CsWO_3_, enhancing their growth mechanism of CsWO_3_.

This decomposition of Au_NR_ into atoms is supported by a previous study [37], where an Au_NR_ suspension was subjected to solvothermal conditions without any precursors. This resulted in the formation of gold flakes, evidenced by the disappearance of the characteristic absorption peaks of Au_NR_ in UV–Vis/NIR spectroscopy. Similarly, the growth enhancement of CsWO_3_ nanorods during the solvothermal process is supported by another study [38], where dissolved Cobalt ions were incorporated at the end of iron oxide (FeOOH) nanorods during solvothermal process. The presence of cobalt ions during the growth process resulted in an increase in the length of the FeOOH nanorods.

Additionally, at sufficiently high Au concentrations, Au atoms tend to recombine with each other, transforming more thermodynamically stable spherical nanoparticles, as depicted in Figure 8b. Specifically, at an Au_NR_ content of 3.0 mol%, the concentration of dissolved Au atoms reached the supersaturation point, leading to the formation of Au atom clusters. Due to the complete occupation of available sites within the CsWO_3_ structure, the excess Au ions underwent recombination, resulting in the formation of independent Au spheres.

**Figure 9:**
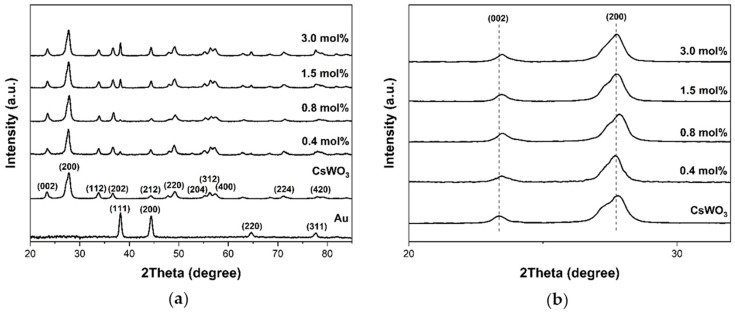
(**a**) XRD patterns of Au_NR_, CsWO_3_, and Au_NR_@CsWO_3_ nanocomposites synthesized by solvothermal mixing method with different Au_NR_ content and (**b**) magnified XRD patterns in the region of 2θ from 20° to 32°.

The XRD pattern in Figure 9 supports the elemental mapping results, indicating that Au atoms can be incorporated into the CsWO_3_ nanorods. No impurities or any new phases were detected in the XRD analysis. However, a closer examination revealed shifting and broadening of the XRD peaks. Comparing the XRD peaks of neat CsWO_3_ and the Au_NR_@CsWO_3_ nanocomposites, the peaks corresponding to the 002 and 200 planes of Au_NR_@CsWO_3_ (at 23.6° and 27.7°, respectively), were slightly shifted toward higher angles. This peak shifting confirms that the Au atoms can be incorporated into a CsWO_3_ nanorod structure without creating any new phases, agreeing with the findings of Venkatesan et al. [39]. They reported similar peak shifts in metal ion-doped V_2_O_5_ samples, indicating the successful incorporation of the metal ion into the pure V_2_O_5_ host structure.

**Figure 10:**
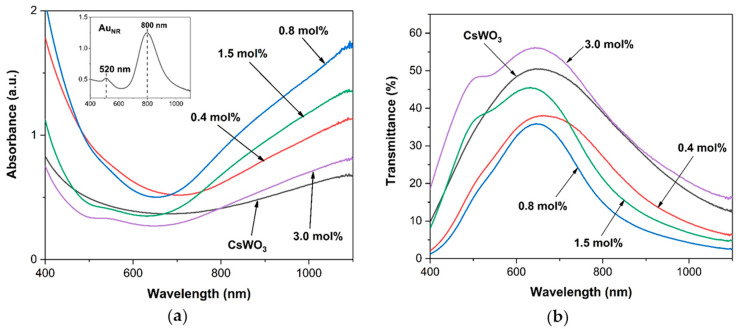
(**a**) Absorbance and (**b**) transmittance spectra of CsWO_3_ nanorods and Au_NR_@CsWO_3_ nanocomposites synthesized by solvothermal method with different Au_NR_ content.

Figure 10 shows the absorbance and transmittance spectra of CsWO_3_ nanorods and the Au_NR_@CsWO_3_ nanocomposites synthesized by the solvothermal method. The incorporation of Au_NR_ had a significant impact on the optical properties of the nanocomposites. The absorption value at a wavelength of 400 nm increased significantly with the addition of 0.4 and 0.8 mol% of Au_NR_. This increase in absorption was due to the growing length of the CsWO_3_ nanorods, which reached up to 4–7 µm after the incorporation of Au. Interestingly, the nanocomposite with 0.8 mol% of Au_NR_ exhibited much higher NIR absorption compared to that with 0.4 mol% of Au_NR_, due to the presence of a larger number of CsWO_3_ in the micrometer size range, as confirmed by the DLS results shown in Figure 6f. However, as the Au_NR_ content was further increased to 1.5 mol% and 3.0 mol%, the absorption in both the visible and NIR regions demonstrated a tendency to decrease, which can be ascribed to the presence of shorter CsWO_3_ rods within the nanocomposites. The nanocomposites with 3.0 mol% of Au_NR_ exhibited the highest transmittance in visible region.

Notably, the absorbance spectrum revealed the presence of a minor peak at 540 nm, indicating the formation of Au nanospheres, which corresponded to the absorption of Au nanospheres. This was supported by the work of Altunbek et al. [40]. This result confirms the presence of excessive dissolved Au atoms, which preferred to accumulate rather than incorporate into the CsWO_3_ nanostructure, resulting in the formation of Au nanospheres.

**Figure 11:**
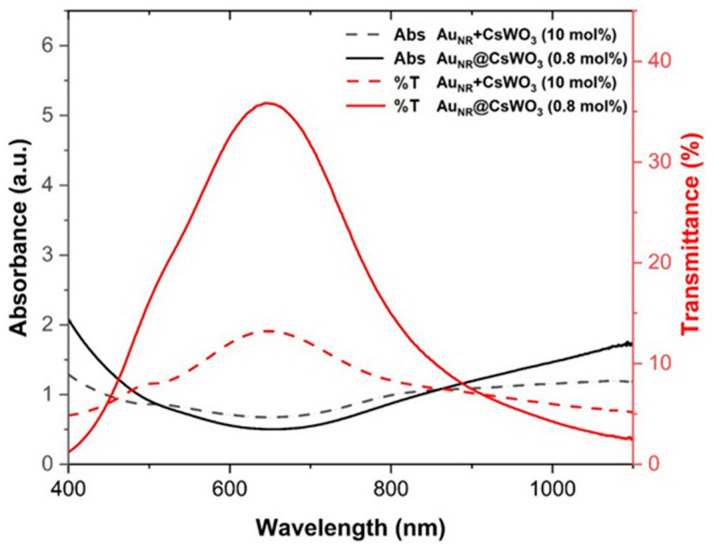
Comparison of absorbance and transmittance spectra between Au_NR_+CsWO_3_ nanocomposites with 10 mol% Au_NR_ and Au_NR_@CsWO_3_ nanocomposites with 0.8 mol% Au_NR_.

According to the results, the solvothermal method enhanced the NIR absorption ability of the nanocomposites compared to those derived from the physical mixing process. Specifically, the solvothermal approach increased the length of the CsWO_3_ nanorods, leading to improved NIR absorption properties. In fact, the nanocomposite synthesized via the solvothermal process with just 0.8 mol% Au_NR_ exhibited superior NIR shielding and visible transmission performance over the composite made by physical mixing with a much higher 10 mol% Au_NR_ content, as shown in Figure 11. Furthermore, the solvothermal process requires fewer steps and a smaller amount of Au_NR_ compared to the physical mixing process. Therefore, the Au_NR_@CsWO_3_ composite materials produced via the solvothermal process are an efficient alternative for use in energy-saving applications that require effective NIR shielding properties.

## 4. Conclusions

Au_NR_/CsWO_3_ nanocomposites were successfully prepared by two different methods: a physical mixing process and a solvothermal process. In the physical mixing approach, the enhancement of NIR absorption of Au_NR_+CsWO_3_ was promoted through the surface plasmon resonance phenomena and the energy coupling between Au and CsWO_3_ components. In contrast, in the solvothermal approach, Au atoms were incorporated into the CsWO_3_ structure, leading to the increase in the longitudinal axis of CsWO_3_ nanorods. The composite prepared by the solvothermal method exhibits better NIR absorption and visible transmission compared to that obtained from the physical mixing process. This latter Au_NR_@CsWO_3_ nanocomposite prepared by the solvothermal method may efficiently be a good candidate in energy-saving window applications.

## Data Availability

The data presented in this study are available on request from the corresponding author. The data are not publicly available due to privacy restrictions.
